# The Role of HIF-1α in Bone Regeneration: A New Direction and Challenge in Bone Tissue Engineering

**DOI:** 10.3390/ijms24098029

**Published:** 2023-04-28

**Authors:** Jiaqian You, Manxuan Liu, Minghui Li, Shaobo Zhai, Sezhen Quni, Lu Zhang, Xiuyu Liu, Kewen Jia, Yidi Zhang, Yanmin Zhou

**Affiliations:** 1 Jilin Provincial Key Laboratory of Tooth Development and Bone Remodeling, Hospital of Stomatology, Jilin University, Changchun 130021, China; youjq21@mails.jlu.edu.cn; 2 School of Stomatology, Jilin University, Changchun 130021, China

**Keywords:** hypoxia-inducible factor, hypoxia, bone regeneration, bone formation, bone tissue engineering

## Abstract

The process of repairing significant bone defects requires the recruitment of a considerable number of cells for osteogenesis-related activities, which implies the consumption of a substantial amount of oxygen and nutrients. Therefore, the limited supply of nutrients and oxygen at the defect site is a vital constraint that affects the regenerative effect, which is closely related to the degree of a well-established vascular network. Hypoxia-inducible factor (HIF-1α), which is an essential transcription factor activated in hypoxic environments, plays a vital role in vascular network construction. HIF-1α, which plays a central role in regulating cartilage and bone formation, induces vascular invasion and differentiation of osteoprogenitor cells to promote and maintain extracellular matrix production by mediating the adaptive response of cells to changes in oxygen levels. However, the application of HIF-1α in bone tissue engineering is still controversial. As such, clarifying the function of HIF-1α in regulating the bone regeneration process is one of the urgent issues that need to be addressed. This review provides insight into the mechanisms of HIF-1α action in bone regeneration and related recent advances. It also describes current strategies for applying hypoxia induction and hypoxia mimicry in bone tissue engineering, providing theoretical support for the use of HIF-1α in establishing a novel and feasible bone repair strategy in clinical settings.

## 1. Introduction

With the increasing aging of the population, the incidence of bone defects caused by cancer, tumors, and trauma is rising, which has increased the need for bone reconstruction in orthopedic surgery [1]. The use of autologous bone grafting remains the “gold standard” for the treatment of bone defects in clinical practice, but the limited availability of donor bone and the risk of donor site infection limits the widespread use of autologous bone grafting in clinics [2,3,4]. Bone tissue engineering is becoming a promising tool due to its ability to overcome several problems, such as insufficient living tissue for transplantation. In recent years, with the growing emphasis on the “bionic design” of bone tissue engineering biomaterials, traditional tissue engineering has shifted from using biomaterials to promote bone regeneration to using biomaterials to generalize the process of embryonic development, which is an approach that was termed “developmental engineering” [5,6]. It is well known that bones develop through two pathways during embryonic development, namely, endochondral ossification (ECO) and intramembranous ossification (IMO) [7]. The repair phase of bone defects includes two stages: the soft callus phase and the hard callus phase [8]. In the soft callus phase, the recruited MSC produces two different forms of differentiation depending on the microenvironment. MSCs on the periosteal surface near the fracture site differentiate into osteoblasts that form woven bone, mainly through IMO, whereas MSCs in the central region of the fracture site where the oxygen content is low differentiate into chondrocytes via ECO. These chondrocytes secrete an extracellular matrix to form a cartilaginous soft callus and eventually regulate mineralization through chondrocyte apoptosis [9]. In the hard callus phase, osteoblasts follow invading blood vessels to the site of calcified cartilage to initiate the production of the hard callus [10,11]. Direct (or primary) bone healing occurs via IMO when the size of the bone defect is minor and the fracture ends of bone defects are tightly connected. Nevertheless, indirect (or two-stage) bone healing occurs via ECO, forming cartilaginous fracture crusts and eventually remodeling into mature bone when the bone defect is significant [12]. However, most current biomaterials for bone defect repair are designed to achieve bone healing via IMO, while strategies for ECO in bone defects are less common. Bone tissue constructed via ECO showed excellent regenerative potential over the past decade. It has dramatically enhanced bone repair, but in treating more significant areas of defective bone tissue, insufficient angiogenesis and limited perfusion capacity led to ischemic necrosis and core degradation [13,14]. Therefore, more attention should be paid to ECO in terms of bionic requirements and the type of healing that occurs during bone repair [14]. It is known that ECO usually occurs in a hypoxic environment during embryonic development. Oxygen-sensitive hypoxia-inducible factor-1α (HIF-1α), which is one of the key transcription factors regulating ECO, can be activated under hypoxia to play an essential regulatory role in the process of hypoxic cellular adaptation [15]. Hypoxia is classified according to the degree of hypoxia as follows: severe hypoxia (1% O_2_), moderate hypoxia (2% O_2_), and mild hypoxia (5% O_2_). When the oxygen tension is >5%, the half-life of HIF-1α is very short (<5 min) [16]. However, HIF-1⍺ is primarily active during short-term hypoxic tension when oxygen is below 1%. HIF-1α can compensate for the reduction in available oxygen caused by reduced oxygen levels by altering the cellular metabolism and activating substances associated with regulating oxygen homeostasis, such as erythropoiesis and the construction of vascular networks. Although HIFs were initially identified in therapeutic approaches to oncological diseases, there was recently an increasing number of studies on the osteogenesis of HIF-1α [17]. However, the specific mechanism of the effect of HIFs on bone tissue production is still not fully confirmed. This review lays a theoretical foundation for the future development of bone tissue engineering by revealing the effects of HIF-1α on cellular activity, vascular construction, and metabolism during bone regeneration.

## 2. Basic Structure and Biological Properties of HIF

HIFs belong to the Per/Arnt/Sim superfamily of basic helix–loop–helix transcription factors, which were shown to play an essential role in vascular–osteogenic coupling during bone repair. HIFs, which are critical proteins that regulate tumors, inflammation, and other related events caused by a hypoxic environment, consist of oxygen-dependent α subunits and β subunits that are not held by oxygen content. There are three α subunits (HIF-1α, HIF-2α, and HIF-3α) and three β subunits (HIF-1β, HIF-2β, and HIF-3β, also known as ARNT1, ARNT2, and ARNT3, respectively) that are known to exist [15,18]. The transcription factors HIF-1α and HIF-2α induce downstream target gene expression by forming heterodimers with the HIF-1β subunit [19]. HIF-1α, whose expression is regulated by oxygen-sensitive proteolytic mechanisms, is constitutively produced at high levels in most cells and has the shortest half-life, whereas HIF-2α is expressed in a more tissue-restricted manner [20,21,22,23]. On the other hand, HIF-3α is expressed only in specific tissues and exerts negative regulation in response to hypoxia by competing with HIF-1α or HIF-2α when binding to target genes [24]. Of the three HIF-⍺ subunits, HIF-1⍺ and HIF-2⍺ are the most studied. HIF-1⍺, which is primarily active in short periods of low oxygen tension (when oxygen is below 1%), is mainly responsible for regulating short-duration hypoxia-induced physiological responses. In comparison, HIF-2⍺ is continuously activated under mild or physiological hypoxia (when oxygen tension is at 5%) and is essential in coordinating hypoxic responses resulting from prolonged hypoxia [25,26,27]. Because prolonged hypoxia adversely impacts bone healing, HIF-1α shows excellent potential in bone defect repair compared with HIF-2α, and this review focused on a detailed summary of the role played by HIF-1α in bone regeneration.

**Figure 1:**
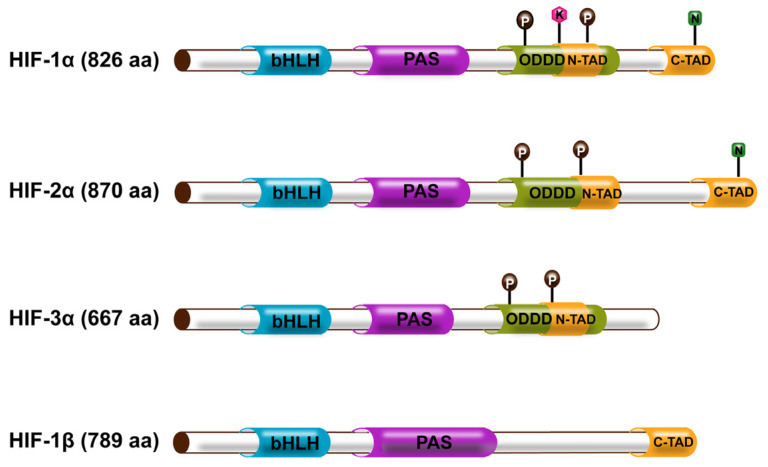
The structure of the HIF family. bHLH and PAS regions act as essential regions for the oxygen-sensitive α subunit and constitutively expressed β subunit that are required for DNA binding and heterodimerization of the α subunit and β subunit. ODDD is an O2-dependent degradation structural domain that primarily regulates proteasomal degradation and rapid ubiquitination of the α-subunit under non-hypoxic conditions. N-TAD or C-TAD at both ends mainly mediate the transcription of hypoxia-activated downstream genes. Both HIF-1α and HIF-2α have two transactivating structural domains, whereas the HIF-3α and HIF-1β subunits have only one TAD. Reproduced with permission from [15]. Copyright (2022) Elsevier.

**Figure 2:**
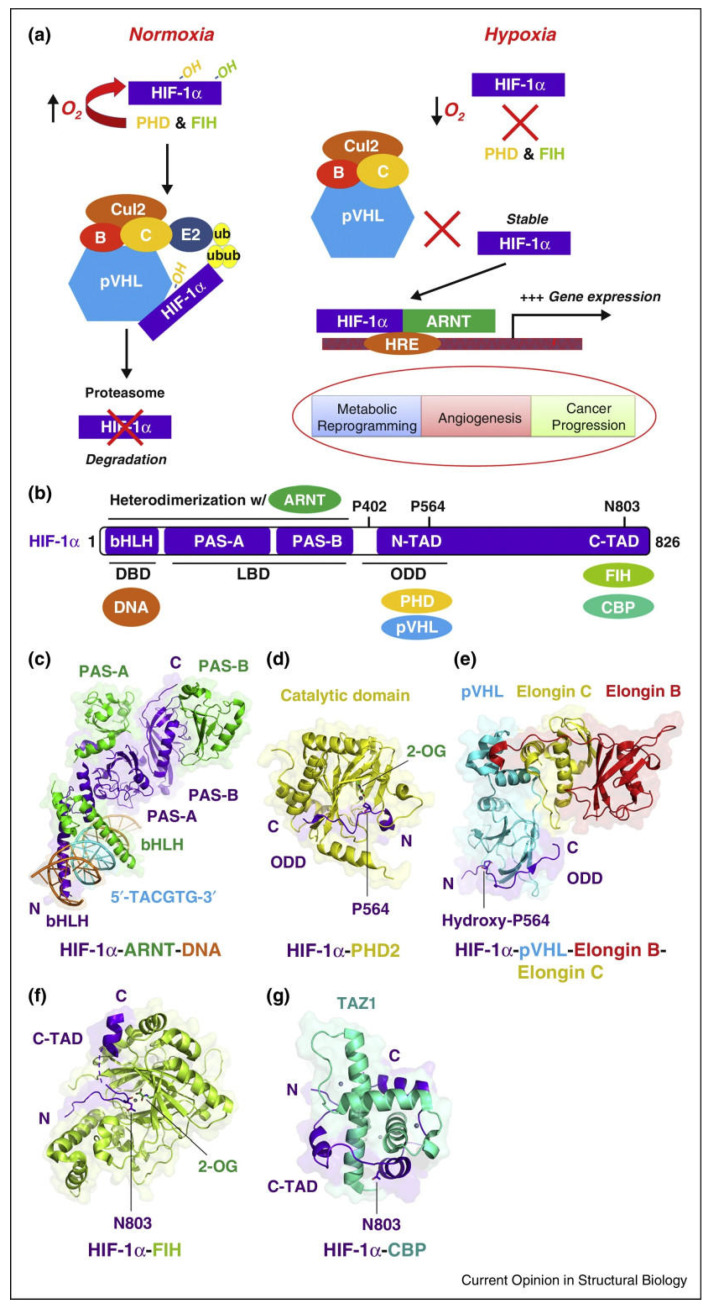
Functional interactions formed between HIF-1α and other macromolecules. (**a**) The oxygen-dependent regulation of HIF-1α-ARNT transactivation by pVHL-mediated HIF-1α protein degradation. PHD: prolyl hydroxylase domain protein; FIH: factor-inhibiting HIF; pVHL: von Hippel–Lindau protein; ub: ubiquitin. (**b**) A diagram of the HIF-1α protein showing the function and interacting macromolecules (DNA or proteins) for each domain. DBD: DNA-binding domain; LBD: ligand-binding domain; ODD: oxygen-dependent degradation domain; TAD: transactivation domain. (**c**) Crystal structure of an HIF-1α-ARNT-DNA complex showing the binding and recognition of the HRE site (5′-TACGTG-3′, colored in cyan) on the DNA by bHLH domains. (**d**) Crystal structure of the catalytic domain of PHD2 complexed with the ODD region of HIF-1α, including a P564 residue. (**e**) Crystal structure of P564-hydroxylated HIF-1α ODD peptide binding to the pVHL–Elongin B–Elongin C complex. (**f**) Crystal structure of FIH in complex with the C-terminal TAD fragment of HIF-1α, including an N803 residue. (**g**) NMR solution structure of the TAZ1 motif of CBP binding to the C-TAD peptide of HIF-1α, including an N803 residue. Reproduced with permission from [18]. Copyright (2017) Elsevier.

**Figure 3:**
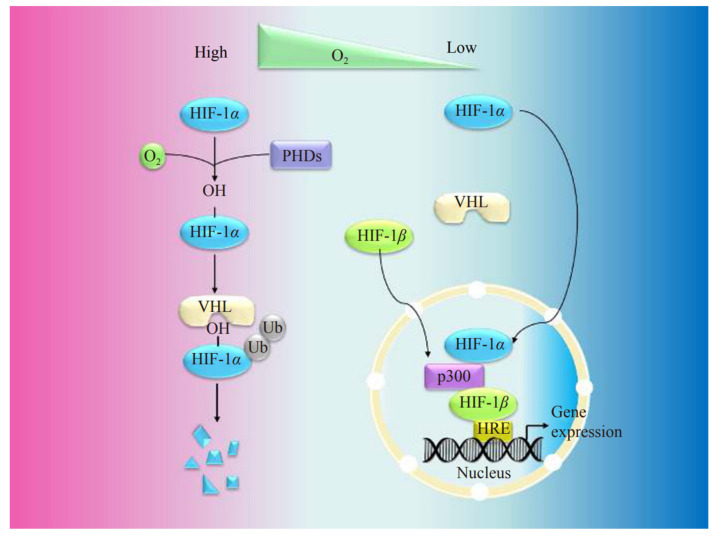
The von Hippel–Lindau tumor suppressor protein (VHL)-dependent HIF-1α signaling pathways. Under normoxia conditions, HIF-1α protein is recognized by prolyl hydroxylase protein (PHD) before combination with a von Hippel–Lindau protein (VHL) and ubiquitination (Ub). It is subsequently degraded by the proteasome. Under hypoxia conditions, PHD is inactivated. HIF-1α and HIF-1β translocate to the nucleus, thus forming a complex with p300 in the nucleus, binding to the hypoxic response element (HRE) and activating gene transcription. Reproduced with permission from [28] Copyright (2021) Elsevier.

Although oxygen content changes the influence of the expression of HIF-1α, HIF-1α does not directly sense the change in oxygen tension. Still, the regulation of HIF-1α expression through the perception of oxygen tension by prolyl hydroxylases (PHD1, PHD2, PHD3) and the asparagine hydroxylase factor inhibiting HIF (FIH) depend on 2-oxoglutarate (2-OG) and Fe^2+^. This specific regulation depends on the particular structural domain of HIF-1α. The structure of HIF-1α contains four main structural domains (shown in Figure 1), including the bHLH-binding structural domain and the tandem-localized PAS structural domain in the N-terminus, which are mainly responsible for the binding of HIF-1α to HIF-2β to form heterodimeric HIF-1 [28]. Then, HIF-1 binds to the target gene to activate the expression of the downstream target protein. The intermediate segment is the oxygen-dependent regulated degradation domain ODDD. When oxygen levels are normal (oxygen tension > 5%), two specific proline residues (P402 and P564, either or both) in the oxygen-dependent degradation domain (ODDD) of HIF-1α are recognized and hydroxylated by PHD [29]. HIF-1α, which is modified by hydroxylation, could be identified further using the Von Hippel–Lindau protein (VHL), which is a component of the E3 ubiquitin ligase complex that modifies HIF-1α. VHL ubiquitinates HIF-1α and recruits the E3 ubiquitin ligase protein complex that targets HIF-1α for degradation. Furthermore, ubiquitinated HIF-1α is then hydrolyzed and subsequently unable to function [30,31]. Finally, there are two transactivation structures, namely, N-TAD and C-TAD, located at the C-terminus. Under a hypoxic environment, C-TAD is responsible for recruiting co-activators, such as CBP/p300, to form a transcriptional complex to regulate HIF-1α gene transcription, while N-TADis mainly responsible for the stable expression of HIF-1α to prevent its degradation [32]. When oxygen levels are normal (oxygen tension > 5%), the asparagine residue (N803) in the C-terminal transcriptional activation domain is hydroxylated by FIH, which prevents the interaction of HIF-1α with transcriptional co-activators (histone acetyltransferase CPB and P300) [33,34]. In a hypoxic environment (oxygen tension < 5%), the activity of PHD and FIH is inhibited by the inability to obtain sufficient oxygen molecules, leading to the accumulation of HIF-1α in the cytoplasm before it successfully enters the nucleus. In the nucleus, HIF-1α binds with HIF-1β through the N-terminal bHLH and PAS domains to form dimeric HIF-1. Heterodimeric HIF-1 subsequently binds to the core HRE sequence (5′-NRCGTG-3′) associated with the target gene in the regulatory region, thereby promoting transcription of the downstream target gene [18,35]. At that time, the HIF transactivation domain (TAD) in the C-terminal segment recruits the transcriptional co-activator CBP/p300 to form a transcriptional complex (shown in Figure 2 and Figure 3). The organism adapts to the hypoxic environment by regulating the processes of glycolysis, erythropoiesis, angiogenesis, cell proliferation, and apoptosis [36]. In addition to the oxygen-dependent regulation of HIF-1α described above, growth factors—such as insulin-like growth factors—and specific environments—such as nitric oxide, nitrogen dioxide, and ROS—may also affect the HIF expression by directly or indirectly regulating PHD or FIH [37].

## 3. Bone Formation

Specifically, the process of natural bone reconstruction was divided into three phases: the initial inflammatory phase, the repair phase, and the remodeling phase [38]. The natural bone possesses various characteristics, such as the ability to spontaneously generate inflammation to clear necrotic tissue, the ability to transition to the repair phase at the appropriate time, desirable mechanical properties, and well-developed vascular and neural networks. Although many innovative biomaterials were developed through bone tissue engineering to create more desirable biomaterials, which usually only mimic certain aspects, few biomaterials can fully imitate the entire bone healing process in all phases. However, current bone tissue engineering seems to put less emphasis on the need for the inflammatory phase and more emphasis on highlighting the speed and number of bone marrow mesenchymal cells (BMSCs) directly induced to differentiate into osteoblasts in a short time, while focusing more on reducing the formation of osteoclasts [39,40,41,42,43,44]. Similarly, during embryonic development, bones are formed by two mechanisms of ossification, namely, IMO and ECO. While we emphasize that IMO shortens the bone-healing time, we seem to forget that ECO could establish a better vascular network and improved mechanical properties, although bone healing via ECO requires more time [6]. How can the advantages of both forms of osteogenesis be applied to biomaterials while minimizing their disadvantages? The answer to this question may be one of the reasons why autologous bone, rather than bone substitutes, has remained the gold standard for bone reconstruction. Therefore, if we want to obtain improved bone regeneration, we must learn from biology [45].

### 3.1. Intramembranous Ossification

**Figure 4:**
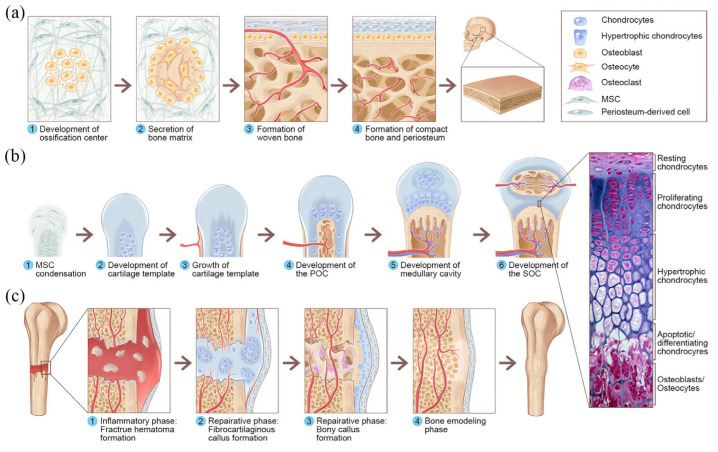
Overview of IMO and ECO during embryonic bone development and fracture healing. (**a**) IMO follows four steps. Step 1: MSCs condense toward the area of future bone formation to form the ossification center, which leads to the development of capillaries and osteoblasts. Step 2: osteoblasts secrete osteoids, which then entrap osteoblasts to transform osteoblasts into osteocytes. Step 3: osteoids secreted around the capillaries result in trabecular matrix formation, while osteoblasts on the spongy bone surface become the periosteum. Step 4: the periosteum then forms compact bone on the surface of the trabecular bone. (**b**) ECO follows six steps during embryonic bone development. Step 1: MSCs are condensed. Step 2: MSCs within the condensed area differentiate into chondrocytes, forming a cartilage template for future long bones, and MSCs surrounding the periphery form the perichondrium. Step 3: chondrocytes in the center of the template develop hypertrophy. Step 4: the hypertrophic chondrocytes secrete angiogenic and osteogenic factors that initiate cartilage matrix mineralization and blood vessel invasion, resulting in the formation of the POC. Step 5: as ossification continues, the epiphysis elongates and the medullary cavity gradually forms. Step 6: following the completion of the above osteogenic activities, the same sequence of events occurs in the epiphyseal regions, leading to the formation of the SOC. (**c**) The healing of fractures follows three phases, namely, the inflammatory phase, the reparative phase, and the remodeling and bone remodeling phase. Reproduced with permission from [50]. Copyright (2021) SAGE Publications.

Compared with the developmental process of ECO, IMO is relatively simple and has been studied more thoroughly, and thus, this review only briefly summarizes IMO. IMO, which is associated with the formation of flat bones, such as the skull, mandible, clavicle, and patella, refers to the direct growth from mesenchymal tissue to bone tissue [46]. The lack of cartilage template formation is one of the significant differences between IMO and ECO. IMO does not involve cartilage templates but relies on the direct differentiation of neural crest cells or mesenchymal precursors into osteoblasts. Embryonic-derived mesenchymal cells gradually accumulate in the area of future bone development, forming a vascularized embryonic connective tissue membrane [47]. At the same time, mesenchymal cells begin to differentiate into osteogenic cells, some of which continue to proliferate into osteoblasts. Osteoblasts begin to secrete an organic intercellular matrix to form osteoids. With the increasing amount of osteoids, the activity of osteoblasts gradually decreases and is encapsulated in the osteoids, which consequently become osteocytes. As the osteoid continues to calcify into the bone matrix, the earliest-appearing bone is formed [48]. The site of the earliest bone formation is called the ossification center, where subsequent osteogenic processes begin and expand in all directions (shown in Figure 4). The skull generally consists of several ossification centers that fuse into a complete bone, where cranial cavity volume grows as the outer layer of bone is continuously produced and the inner layer is continuously resorbed and modified. Recently, Shawon Debnath et al. identified a periosteal stem cell (PSC) in mouse long bones and calvary, which is a physiological precursor of periosteal osteoblasts with traits of clonal pluripotency and self-renewal. It can directly form bone via IMO, providing a new cellular basis for further differentiation of IMO and ECO [49]. 

### 3.2. Endochondral Ossification

ECO is closely associated with the formation of long bones, such as the femur, tibia, humerus, and mandibular condyle. Compared with IMO, ECO is derived from osteoprogenitor cells in the ectoderm during early embryonic development when the fetal growth plate is not yet invaded by blood vessels. ECO begins with mesenchymal stem cells, which first agglomerate in an avascular area in the center of the embryonic growth plate and gradually differentiate into chondrocytes [7,51]. Chondrocytes continue to proliferate and secrete an extracellular matrix rich in type II collagen, thus forming the cartilage template where the future cartilage matrix is deposited [13,52]. As the growth proceeds, the chondrocytes in the center of the original cartilage template stop proliferating and differentiate into hypertrophic chondrocytes, which begin to secrete type X collagen, osteopontin, matrix metalloproteinase-9, matrix metalloproteinase-13, and vascular endothelial growth factor (VEGF) to gradually mineralize the surrounding bone matrix [13,53,54,55,56]. As the cartilage template expands, the tissues continue to consume the oxygen and nutrients provided by diffusion, while the central cardiovascular network of the cartilage template has not yet invaded. Therefore, the oxygen content of the center of the cartilage template gradually decreases and is in a hypoxic state with the establishment of an oxygen concentration gradient, which is the primary stimulus for embryonic cell differentiation and migration [57]. Oxygen, which is one of the primary conditions necessary for cellular physiological activities, poses a significant threat to cell survival during hypoxia. At this point, the hypoxic state in the center of the cartilage template initiates “cell rescue” by driving the expression of HIF-1α. First, HIF-1α induces chondrocytes within the growth plate to secrete VEGF, which increases the input of oxygen and nutrients by inducing the invasion of blood vessels [58]. VEGF, which is one of the vital target proteins of HIF-1α, is also an essential protein in the construction of the entire vascular network, which guides the growth of blood vessels to the area rich in VEGF by attracting tip cells in the endothelium [58]. Second, HIF-1α also activates genes that switch cells to anaerobic metabolic pathways, activating the anaerobic glycolytic pathway, which minimizes oxygen consumption and produces the ATP necessary for survival, contributing to cell survival in a VEGF-independent manner [59]. At the same time, mesenchymal cells in the outer layer of the cartilage template begin to differentiate into osteoprogenitor cells. These two mechanisms are present simultaneously to ensure the establishment of the developing vascular system [60]. The invading vessels bring non-differentiated mesenchymal cells around the periosteum to the mineralization frontier, which subsequently differentiate into osteoblasts and participate in osteogenesis. Bone formation is closely related to vascularization in both space and time, and thus, this concept is now referred to as angiogenesis–ossification coupling, which is, of course, also applicable to similar phenomena in other osteogenic patterns [61,62]. It was shown that stable expression of HIF-1α in endothelial cells can promote the production of more H-type vessels and Osterix-expressing cells [63]. It was already identified that H-type vessels could tightly regulate the coupling of osteogenesis and angiogenesis in the long bones of human beings and mice. Osterix, which is a transcription factor associated with osteoblast differentiation and bone formation, is specifically only expressed in growing bone tissue, which can positively regulate VEGF expression by binding to the VEGF promoter. As the blood vessels around the cartilage template gradually invade the center of the template where hypertrophic chondrocytes accumulate, the Osterix-positive osteoblasts and osteoclasts that were originally around the blood vessels gradually enter the cartilage template along with the blood vessels. They begin to degrade the cartilage to provide space for the osteoblasts to form bone trabeculae and bone marrow hematopoietic cells to form the bone marrow cavity [63,64]. Subsequently, hypertrophic chondrocytes present in the cartilage template begin to die or partly differentiate into osteoblasts [65,66]. This series of cellular activities forms the primary ossification center (POC), which is responsible for the growth of the backbone, while the ends that have not yet been invaded by blood vessels form the epiphysis. The epiphyseal plate can be divided into three regions: the reserve zone, the proliferative zone, and the hypertrophic zone. The reserve zone is the area nearest the epiphyseal end of the plate, and this area contains smaller chondrocytes, which do not participate in bone growth but rather anchor the epiphyseal plate to the bone tissue of the epiphysis. The proliferative zone is the area below the reserve zone closer to the diaphysis that contains a bunch of slightly larger chondrocytes. It produces new chondrocytes via mitosis to replace those that die at the diaphyseal end of the plate. Below the proliferative zone is the hypertrophic zone where the chondrocytes are more mature and larger than those in the proliferative zone. The chondrocytes that maintain growth secretion between the epiphysis and the emerging diaphysis gradually form an expanded epiphyseal plate to further regulate the longitudinal growth of long bones. The chondrocytes continue to secrete an extracellular matrix, which contributes to the expansion of the epiphysis. The calcified matrix zone is the area closest to the epiphysis. Most of the chondrocytes existing in this area have died as the surrounding matrix has calcified, limiting the diffusion of nutrients (shown in Figure 4). However, chondrocytes in the center begin to be exposed to a hypoxic environment because the epiphysis is not yet vascularized. Similar to the formation of the POC, the hypoxic chondrocytes begin to secrete VEGF under the regulation of HIF-1α to induce the continuous entry of blood vessels. At the same time, osteoclasts and osteoblasts also invade, gradually forming the secondary ossification center (SOC) in the epiphysis [67,68]. As the POC and SOC continue to ossify, the epiphyseal plate between them shrinks until the cartilage of the epiphyseal plate is ossified to fuse the backbone and epiphysis. At this point, the length of the bone no longer increases (shown in Figure 4).

Stable expression of HIF-1α during ECO is indispensable for maintaining cartilage morphology [69,70]. Due to the hypoxic microenvironment formed in regions of bone mesenchymal condensation within the fetal cartilage template, HIF-1α expression is significantly upregulated in those sites. During development, knockdown of HIF-1α causes chondrocyte death, but knockdown of the enzyme required for HIF-1α degradation promotes chondrocyte proliferation. This may be attributed to the fact that upregulation of HIF-1α activates the expression of the transcriptional regulator SRY-related high-mobility group box gene 9 (Sox-9) and promotes the expression of downstream target genes, including aggrecan and type II collagen, which induces the differentiation of BMSC and ADSC into chondrocytes [71,72,73,74]. Studies showed that HIF-1α can induce the differentiation of MSCs into chondrocytes to promote chondrogenesis by activating the expression of Sox [75]. HIF-1α expression is not limited to hypoxic culture environments, and it was shown that engineered murine MSCs stably expressing HIF-1α under normoxic conditions can enhance BMP2-induced chondrogenic differentiation [76,77]. Indeed, the expression of HIF-1α downstream proteins can be regulated even under normal oxygen content culture conditions, as long as the transplanted biomaterials can spatially control the stable expression of HIF-1α in the cells.

Bernard et al. used a critical bone defect model to compare the differences in bone regeneration between ECO and IMO, which found that ECO produced new bone with better mechanical properties similar to cortical bone and was able to more aggressively induce the construction of vascular networks at an early stage [78]. However, IMO makes bone that undergoes a more intense inflammatory resorption and regeneration process, resulting in bone more similar to cancellous bone. The study also found that the ECO pathway first initiates bone tissue regeneration in the center of the defect, while the IMO pathway, where the bone tissue is more dependent on the supporting of the bio-scaffold surface, exhibits osteogenesis that first follows the contours of the scaffold surface. Another part of the study showed that in minor bone defects, due to reduced movement between the surfaces of the broken edges, this kind of bone healing process is more similar to IMO. When the defect area is significant enough that movement between the surfaces of the two bone breaks occurs, the wound healing pathway resembles the ECO pathway. In summary, IMO has a faster rate of osteogenesis than ECO but has difficulty expanding the bone size. Hence, IMO is more suitable for minor or critical-sized bone defects that do not require much vascularization. However, ECO offers an up-and-coming solution for the reconstruction of significant or inevitably displaced bone defects due to the formation of improved mechanical strength and a vascular network by ECO to prevent central bone necrosis [78].

## 4. The Role of HIF-1α in Bone Formation 

**Figure 5:**
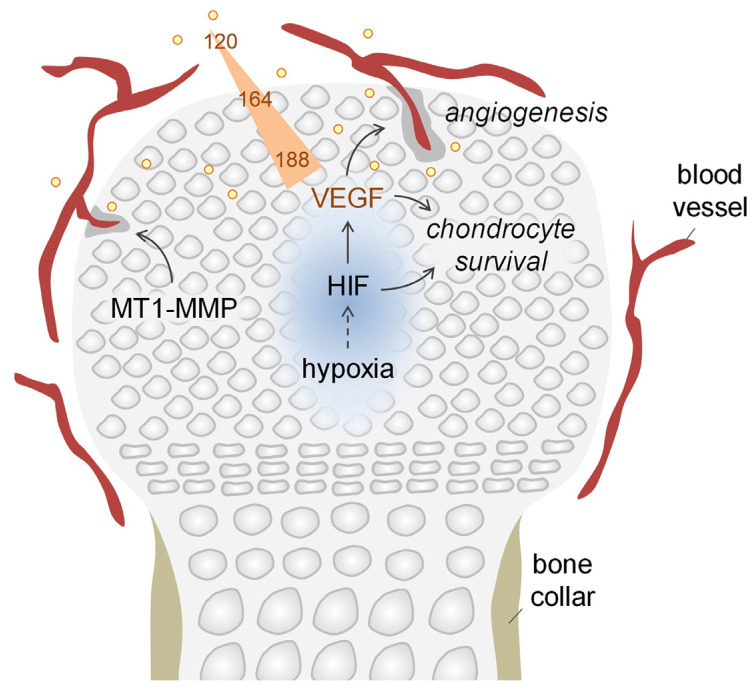
As blood vessels have not yet formed, the center of the growth plate becomes hypoxic due to the lack of oxygen delivery, which stabilizes the expression of HIF-1α. HIF-1α promotes the production of VEGF, which is essential for the diffusion of oxygen to the border of the epiphyseal plate in order to stimulate the sprouting of blood vessels in the surrounding epiphyseal plate. At the same time, HIF-1α enables chondrocytes to survive in a hypoxic environment by altering their metabolism. In addition to HIF-induced angiogenesis, the production of epiphyseal vessels is regulated by MT1-MMP. Reproduced with permission from [58] Copyright (2017) Elsevier.

In the last two decades, bone tissue engineering has developed so rapidly that bone repair has been dramatically facilitated by transplanting a series of osteogenic elements into the bone defect area using biomaterials, such as cytokines and proteins, that induce osteogenic differentiation of mesenchymal stem cells or direct implantation of BMSC-containing biomaterials [79,80,81,82,83,84,85]. However, most biomaterials are designed to follow IMO, which seems to encounter certain obstacles in the treatment of significant bone defects caused by trauma, bone tumors, infections, and so on. Failure to form an excellent vascular network within the bone tissue formed by IMO leads to ischemic necrosis and degradation in the central region of the material, which limits the clinical translation of the material in bone tissue engineering. In contrast, ECO is considered to be a critical tool for addressing this defect. Hypertrophic chondrocytes during cartilage osteogenesis can induce the production of a series of cytokines, such as VEGF, EGF, PDGF, and SDF, which leads to the construction of a vascular network during the induction of bone formation and undoubtedly paves a sufficient vascular network for the repair of significant bone defects (shown in Figure 5) [86]. In recent years, there was an increasing number of investigations on the design of biomaterials based on ECO. Some studies showed that ECO via controlled implantation of biomaterials to induce chondrogenesis achieved better osteogenic results than IMO for the repair of significant bone defects [78,87]. As a critical regulator in cartilage osteogenesis, HIF can play an essential role in bone repair by inducing vascular invasion through oxygen concentration gradients and altering cellular metabolism.

### 4.1. Promotion of Angiogenesis

The collaborative development of bone matrix mineralization and vascular network construction are two crucial factors in the repair of bone defects [58,63]. The failures of biomaterials to successfully construct vascular networks often result in a lack of osseointegration and internal necrosis of the tissue, which severely limits the treatment of significant bone defects and the development of larger bone grafts [13,88,89,90]. Without a well-developed vascular network at the site of the bone defect, the cells surrounding the graft have to rely on diffusion pathways to absorb nutrients and remove metabolic products. However, the distance of nutrient diffusion in the body is usually limited to a few hundred microns, and thus, the diffusion pathway alone cannot support the nutrient and oxygen requirements of the cells during the entire bone healing process. The construction of the vascular network responsible for the transport of oxygen, nutrients, and metabolites during bone regeneration consequently largely determines the final fate of the biomaterials. Several strategies were designed to improve vascularization in bone tissue regeneration, such as the incorporation of pro-angiogenic factors, such as VEGF, “bio-coating” of scaffolds, and gene therapy [91,92,93,94,95,96]. However, HIF-1α, which is one of the critical factors in the construction of vascularization during growth and development, has not been adequately considered in the design of bone tissue engineering.

In the skeleton, a single vascular network system consists of two main types of capillaries. L-type vessels, which are mainly located in the diaphysis and confluent to the central vein, are weakly positive or negative for the expression of two endothelial cell marker antibodies (platelet endothelial cell adhesion molecule-1 (CD31), endomucin) [58]. H-type vessels, located mainly in the metaphysis and subperiosteum, which are strongly positive for the expression of CD31 and endomucin, are connected to arteries with a high proliferative capacity [97,98,99]. Although H-type vascular endothelial cells constitute a low proportion of bone endothelial cells and total bone marrow (1.77%), they selectively recruit most of the Osterix-positive osteoprogenitor cells (70 ± 1.9%), type I α-collagen-positive osteoblasts (74 ± 3.3%), and Runx2-positive osteoprogenitor cells (82.63 ± 1.8%) from the bone tissue to vascularization [63,98,100]. At the same time, H-type vessels express high levels of osteogenic factors, such as bone morphogenetic proteins (BMPs), fibroblast growth factors (FGFs), and platelet-derived growth factors (PDGFs), which regulate the activity of osteoblasts and chondrocytes, thereby ultimately combining angiogenesis and osteogenesis. Increased vascularity may recruit large numbers of osteoblasts and osteoclasts, secreting osteogenic growth factors, such as BMP, which contribute to osteoblast maturation and differentiation, as well as bone formation. H-type vessels secrete VEGF and simultaneously regulate the osteogenic activity of perivascular osteoblasts, which successfully combine angiogenesis and osteogenesis. The current study showed that HIF-1α is a critical protein in regulating H-type angiogenesis. When HIF-1α accumulates to a certain level, it induces the recruitment of osteogenic progenitor cells by inducing H-type angiogenesis, which further links angiogenesis and osteogenesis [63,101]. Mice that are deficient in HIF-1α have a significant reduction in angiogenesis and bone formation [100,102,103]. Wei Lu et al. co-cultured human umbilic vein endothelial cells (HUVECs) with Sr-encapsulated micro/nanotitanium (SLA-Sr) extracts, which showed that increased H-type vessel formation was observed around the SLA-Sr implants, and early vascularization formation significantly improved osseointegration [81]. These results may be related to the fact that Sr promotes the stable expression of HIF-1α by activating the Erk1/2 signaling pathway. Zhang et al. further verified that adding Sr to the surface of nanoporous titanium implants could promote increased phosphorylation of Erk1/2 to enhance the expression of HIF-1α, which leads to the coupling of angiogenesis and osteogenesis and ultimately yields rapid vascularized osseointegration [104]. In addition to HIF-1α-induced angiogenesis, membrane type-1 matrix metalloproteinase (MT1-MMP)-mediated cartilage degradation also promoted epiphyseal vessel growth. On the other hand, the hypoxic environment indirectly promotes the expression of downstream SDF-1/CXCR4 axis and VEGF, which was identified as one of the essential factors for angiogenesis through the upregulation of HIF-1α expression, while stromal-cell-derived factor 1 (SDF-1) and its receptor CXC chemokine receptor 4 (CXCR4) are mainly responsible for mobilizing circulating bone-marrow-derived stem cells and endothelial progenitor cells to the site of ischemia and promoting microangiogenesis at the site of bone injury [100,103,105,106,107,108,109]. HIF-1α stimulates the secretion of VEGF in BMSCs through angiopoietin-1 (Ang-1) and angiopoietin-2 (Ang-2) to mediate osteogenesis and angiogenesis, which constitute a reciprocal regulatory relationship known as the “HIF-1α-VEGF-Ang-1 axis” [110,111]. Systemic administration of SDF-1 effectively mobilizes bone-marrow-derived endothelial progenitor cells to recruit to the area of ischemia and participate in constructing the vascular network at that site [112]. However, although VEGF has been considered the primary regulatory mediator of the angiogenic effect of HIF, the role of erythropoietin (EPO) as one of the downstream target genes of HIF in promoting angiogenesis is not neglected [113]. Yu et al. showed that EPO encourages the metabolism of erythrocytes and angiogenesis by promoting the mitosis of endothelial cells, which increases the oxygen-carrying capacity of blood to assist cells in adapting to a hypoxic environment (shown in Figure 5) [114].

### 4.2. Promotion of Osteogenesis

**Figure 6:**
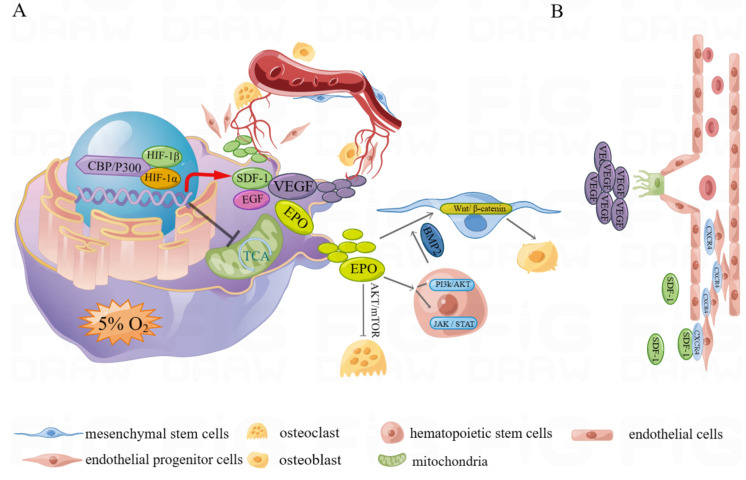
(**A**) The hypoxic environment stabilizes the expression of HIF-1α, which promotes angiogenesis–osteogenesis coupling by promoting the expression of osteogenic and angiogenesis-related proteins. At the same time, the metabolic pattern is altered through the inhibition of the tricarboxylic acid cycle to synergistically promote osteogenesis. (**B**) The stable expression of HIF-1α promotes the production of VEGF and SDF-1, in which VEGF induces vascular growth toward the target area by inducing endothelial cell and tip cell sprouting and migration; SDF-1 recruits circulating endothelial progenitor cells through CXCR4 to promote angiogenesis.

For the repair of significant bone defects, bone substitutes usually exhibit poor therapeutic ability, which may be related to the fact that the repair process of significant bone defects involves ECO [115,116]. In contrast, existing bone substitutes cannot successfully initiate endogenous bone regeneration, which refers to the ability to activate the recruitment of stem cells involved in ECO and the formation of cartilage intermediates. Recently, Dan Lin et al. proposed the chondrogenic/osteogenic balance hypothesis, which states that two equally necessary conditions are required for the rapid initiation of regeneration and subsequent bone formation, namely, chondrogenic induction at an early stage and osteogenic induction at a later stage [79]. The stable expression of HIF-1α as one of the necessary conditions for ECO plays a vital role in the induction of chondrocyte hypertrophy and initiates the transition from the cartilaginous phase to osteogenesis. However, the current debate in this area focuses on the “determination of the optimal time for early cartilage induction to late osteogenesis induction.” A recent study showed that activation of the HIF signaling pathway on day 5 after implant placement and the induction of cartilage-to-bone conversion on day 7 could be designed to achieve optimal bone regeneration rates in vivo [80]. More experiments may be needed in the future to determine the timing of this conversion. In addition, EPO, which is one of the significant target proteins regulated downstream by HIF-1α, was shown to stimulate osteogenesis. Kim et al. showed that EPO induces BMP2 production through the activation of the JAK/STAT and PI3k/AKT signaling pathways in hematopoietic stem cells (HSCs), which indirectly promotes the differentiation of MSCs into osteoblasts (shown in Figure 6) [117,118]. Zheng et al. found that EPO promotes the expression of osteogenic proteins, such as Runx2, Osterix, and Osteocalcin, as well as alkaline phosphatase activity and calcium deposition through the Wnt/β-linked protein signaling pathway in a dose-dependent manner [119], which enhances the osteogenic activity of human periodontal ligament stem cells. Meanwhile, Chen et al. sustained the stable expression of HIF-1α via VHL gene knockdown, which showed that stable expression of HIF-1α promotes the osteogenic differentiation of BMSCs while inhibiting its differentiation to osteoblasts [120]. More interestingly, Shao et al. found that the effect of HIF-1α on osteogenic activity is also influenced by age. P53, as one of the biomarkers of aging, acts as an intermediate regulator of HIF-1α on the expression of vascular and osteogenic-related genes, which inhibits osteogenic and vascular activity by suppressing the expression of osteogenic-related proteins, as well as VEGF [121]. Shao et al. showed that HIF-1α achieved osteogenesis-promoting effects by inhibiting p53 activity in young 2-month-old mice, whereas HIF-1α activated p53 in aging 18-month-old mice to negatively affect osteogenesis and angiogenesis owing to the accumulation of ROS that reverses the relationship between HIF-1α and p53. It is well known that bone marrow is chronically exposed to a hypoxic microenvironment, which plays an essential role in maintaining the function and stemness of BMSCs. Several related studies have investigated the different effects of oxygen concentration and hypoxia duration on bone repair. Kuss et al. showed that cells treated with long-term hypoxia (21 days) showed a significant decrease in cell activity and a potent inhibition of angiogenesis compared with the short-term hypoxia treatment group (7 days) [122]. Nicolaije et al. demonstrated that the hypoxic environment has different effects on osteoblasts at various stages of osteogenic differentiation in the early stages of osteogenesis. The proliferation of osteoblasts is highest when exposed to 20% oxygen, while in the later stages, 2% oxygen is most favorable for osteoblast proliferation [123]. Some studies showed that the senescence of BMSCs is inhibited and the efficiency of osteogenic differentiation is increased when the oxygen content is 5% [124,125]. When the oxygen content is 1–2%, the altered expression of HIF-1α in BMSCs activates the Notch1 and ERK/p38/MAPK signaling pathways, which inhibits the osteogenic differentiation of BMSCs [126,127]. In addition, the stable expression of HIF-1α promotes adipogenic differentiation, but not osteogenic differentiation, of BMSCs when the oxygen content is below 0.2% [128]. Costa et al. showed that HIF-1α, which acts as a potent regulator of bone tissue engineering, promotes the proliferation and osteogenic differentiation of BMSCs [129]. Still, some contrary experimental results showed that stable expression of HIF under hypoxic conditions prolongs the cell cycle of BMSCs, reduces their proliferative capacity, and hinders their differentiation into osteoblasts. Other studies showed that RANKL expression in osteoblasts is promoted by activators of the transcription 3 (STAT3) pathway and Janus kinase 2 (JAK2) signal transducers, which induces the differentiation of BMSCs into osteoclasts, leading to osteoporosis [130,131,132]. In addition, in vivo studies showed that hypoxia can be a potential treatment strategy for osteonecrosis [133]. Hypoxia promotes bone regeneration in osteonecrosis of the femoral head through the HIF-1α/β-catenin pathway [134]. Thus, although adaptive physiological responses induced by hypoxic environments were studied more intensively in recent years, the effects of hypoxic environments on bone repair are still highly controversial. Some conflicting experimental results may be related to the degree, duration, and frequency of hypoxic exposure and the age of the subjects, hence more attention should be paid to the uniformity of these conditions in future studies that explore the relationship between hypoxia and osteogenesis.

In addition, HIF-1α seems to affect bone repair through osteoimmunity, but the exact effects seem to be somewhat controversial [93,135]. Hirai et al. showed that activation of HIF-1α can downregulate bone-resorption-related factors in the nuclear factor-κB (NF-KB) signaling pathway, and at the same time, stable expression of HIF-1α can specifically inhibit the differentiation of lipopolysaccharide-stimulated macrophages into the M1 phenotype (an inflammation-promoting macrophage phenotype) and increase the production of M2 macrophages (an inflammation-promoting macrophage phenotype), which inhibits bone resorption [135,136]. However, other studies demonstrated that stable expression of HIF promotes the formation of M1-type macrophages [93]. In addition, methods of stabilizing the expression of HIF-1α are diverse, and achieving a stable expression of HIF-1α by different means or drugs also seems to have different effects on bone regeneration. For example, Kimito Hirai et al. used dimethyloxalylglycine (DMOG) and adenovirus-induced constitutively active HIF-1α (CA-HIF1α) to stabilize HIF-1α expression [135]. The CA-HIF1α group enhanced the activation of the NF-κB signaling pathway and subsequent pro-inflammatory cytokine production in LPS-stimulated macrophages. In contrast, the DMOG group exhibited anti-inflammatory effects by downregulating inflammatory factors in the NF-κB signaling pathway in both in vitro and in vivo experiments, which is consistent with previous results showing that DMOG inhibits IL-1β-induced NF-κB activation in vitro and in vivo. Although both CA-HIF1α and DMOG indeed stabilize HIF-1α, functional differences appear to depend partly on the stabilization mechanism, which may also be one of the reasons why studies on HIF-1α in osteogenesis derived conflicting results.

### 4.3. Change in Metabolism 

Recent studies on the role of HIF-1α in the regulation of cellular metabolism have attracted increasing interest [37,137,138,139]. Stegen et al. showed that stable expression of HIF-1α improves cell survival, not exclusively through enhanced angiogenesis, but also through the adaptation of changes in the cellular metabolism of glutamine and glucose, which are required for redox and energy homeostasis [138]. Under normoxic conditions, cells usually prefer aerobic glycolysis in the mitochondria, which produces 36 ATP molecules per molecule of glucose. This metabolic approach oxidizes glucose to pyruvate and produces ATP using the TCA cycle and OxPhos, which consume approximately 90% of the available oxygen [140]. Under hypoxic conditions, HIF-1α, which is considered the primary driver of metabolic adaptation to hypoxia, adjusts the mode of ATP production by regulating cellular energy metabolism to allow cells and tissues to survive when oxygen levels are limited [22]. When HIF-1α is stably expressed, cellular metabolism usually prefers anaerobic glycolysis, in which the TCA cycle and OxPho are suspended to reduce oxygen consumption in the mitochondria and glucose is reduced to lactate rather than pyruvate, rather than aerobic glycolysis under normoxia conditions [137]. This process produces only two counts of ATP per molecule of glucose, but thanks to its rapid production rate, it may produce ATP levels similar to those via aerobic phosphorylation. This fast production of ATP in a hypoxic environment seems to be particularly important for rapidly proliferating cells [37,137]. Endothelial cells during angiogenesis, as well as osteoblasts during the early stages of bone healing when the bone defect site is hypoxic due to early hematoma, behave similarly to immune cells during an immune response. Anaerobic glycolysis promotes the survival and phagocytosis of neutrophils, as well as the formation of neutrophil extracellular traps, which enhances innate immune activity to ensure the successful completion of the inflammatory phase [141]. Cui et al. developed ultrathin 2D titanium carbide MXene (Ti_3_C_2_Tx), which activates the Wnt/β-linked protein signaling pathway by enhancing Wnt–Frizzled complex binding, thereby stabilizing HIF-1α and altering the metabolic reprogramming to anaerobic glycolysis [142]. Cui et al. also found that the activation of anaerobic glycolysis is necessary for the high proliferation of osteoblasts in a pseudo-hypoxic state at the beginning of osteogenesis and the subsequent bone-matrix protein biosynthesis. This may be related to the physiological presence of MSCs within the bone marrow niche in a hypoxic environment. In addition, some studies suggested that lactate can be a potential driver of bone repair. Lactate promotes the recruitment of MSCs and endothelial cells to sites of inflammation and activates the pro-angiogenic NF-κB/IL-8 pathway to drive the expression of cytokines and growth factors, such as VEGF and TGF-β [143,144,145]. Meanwhile, lactate also promotes the resolution of inflammation and the induction of angiogenesis by inducing the production of anti-inflammatory and pro-angiogenic immune cell phenotypes, such as the formation of M2 macrophages [146,147,148]. At the same time, lactic acid not only induces metalloproteinase and collagen synthesis through autocrine and paracrine modes but also increases the rate of proteoglycan gene transcription and post-translational modification of proteins, which drive the deposition of fibrocartilage to promote extracellular matrix formation and bone tissue regeneration [137]. Furthermore, HIF-1α is stably expressed early in the differentiation of BMSCs to maintain the energy required for BMSC survival through glycolysis, but HIF-1α expression is downregulated at later stages to enhance osteogenesis and chondrogenesis induction through aerobic phosphorylation. These results partly explain the conflicting findings regarding HIF-1α generated in different studies concerning bone regeneration [149,150].

## 5. Method of Stabilizing HIF in Bone Tissue Engineering

### 5.1. Gene Therapy

With the development of science and technology, genetic engineering has been gradually applied in the field of bone tissue engineering [151,152]. Genetic engineering, also known as gene-splicing technology or DNA recombination technology, is a complex technology that allows a gene to be replicated, transcribed, and translated in the recipient cell through in vitro recombination by manipulating the gene at the molecular level [153]. Knockout, which is a branch of genetic engineering, is an exogenous DNA introduction technique in which a DNA fragment containing a known sequence is homologously recombined with a gene of the same or similar sequence and incorporated into the genome of the recipient cell to be introduced into the tissue for expression. It is a means to investigate the biological function of a specific gene by disabling the function of the gene in the organism. Three common vectors that can introduce isolated or synthetic genes into recipient cells are plasmids, phages, and animal viruses. The expression of HIF-1α is negatively correlated with the expression of the von Hippel–Lindau (VHL) protein and PHDs, the latter of which can be classified into PHD1, PHD2, and PHD3 phenotypes. Therefore, inactivating the VHL protein and PHDs to stabilize the expression of HIF-1α has become an effective method of genetic engineering. Wu et al. constructed recombinant osteoblasts using Cre-loxP-mediated specific inactivation of Phd1, Phd2, and Phd3 to generate osteogenic progenitor cells with combined genetic inactivation of single or multiple oxygen-sensitive PHD1–3 [154]. Wu et al. also explored the relationship between osteogenesis and the stable expression of HIF-1α caused by the deletion of different PHD types in mice with specific PHD knockouts. It was found that single PHD subtypes and combined inactivation of the PHD1 and PHD3 subtypes appeared to have little effect on bone regeneration effects, but simultaneous inactivation of all three PHD subtypes, combined inactivation of PHD1 and PHD2 subtypes, and combined inactivation of PHD2 and PHD3 subtypes promoted bone regeneration to a large extent. The effect of HIF-1α on vascular regeneration showed a similar pattern, with neither a single PHD subtype nor double co-inactivation appearing to promote vascular regeneration, promoting only simultaneous inactivation of all three subtypes, which showed a vascular regeneration-promoting effect. This suggests that the function of HIF-1α in promoting osteogenesis and vascular regeneration may be related to the specific way in which HIF-1α is stabilized. Feng et al. disrupted VHL gene expression in CD34 hematopoietic stem cells and progenitor cells via transfection with a ribonucleoprotein consisting of Cas9 and VHL-targeting guide RNA, which showed that loss of the von Hippel–Lindau (VHL) protein promoted stable expression of HIF-1α and activated the expression of downstream signaling pathway proteins to mediate adaptive responses [155]. In addition to regulating gene expression through the mutation of the target gene is a type of gene therapy used to stabilize HIF-1α. Deng et al. developed a mutant form of HIF-1α (HIF-1α-AA) that is more stable than wild-type HIF-1α, achieving stable expression of HIF-1α under normoxia by transfecting HIF-1α-AA with an RGD-modified gene vector based on a hyperbranched cationic polymer (RGD-DMAPA-Amyp/HIF-1α-AA) [156]. It was confirmed that RGD-DMAPA-Amyp/HIF-1α-AA is a safe non-viral gene vector with good biocompatibility and high cellular uptake, which can be well endocytosed by human cells. However, the main drawback of current gene therapy is the low efficiency of gene delivery and the problem of nonspecific side effects. Heun et al. developed a magnetically targeted gene transfer technique based on a magnetic nanoparticle–lentivirus (MNP-LV) complex, allowing for site-specific gene delivery to the target [157]. Heun et al. also used this technique to control the HIF-1α-dependent angiogenic healing response in vivo via site-specific regulation of the tyrosine phosphatase activity of SHP-2. These results suggest that SHP-2 could protect HIF-1α transcriptional activity in vitro and in vivo by activating Src to inhibit HIF-1α degradation.

Short interfering RNA (siRNA) is a segment of double-stranded RNA containing 21 to 25 nucleotides that can be introduced to target and regulate the expression of multiple downstream pathways or factors, leading to simultaneous guidance of numerous and interconnected cells [158,159,160,161]. The essence of the process is gene silencing, which prevents translation of the target gene by interfering with the expression of mRNA that is degraded after transcription of a specific gene with a complementary nucleotide sequence. It was shown that HIF-1α expression can be regulated to direct bone formation by siRNA against prolyl-hydroxylase-domain-containing protein 2 (siPHD2) [162]. The gene-silencing approach allows us to control the proliferation and apoptosis of relevant cells by targeting the expression of multiple proteins through a single siRNA introduction. However, in the application of siRNA, we should not only focus on the type, timing, concentration, and number of siRNAs introduced to the receptor but also consider the number of siRNAs that are continuously released over time to avoid taking effect at the wrong time and excessive upregulation of target protein expression. In addition, microRNA (miRNA) is a class of evolutionarily highly conserved non-coding small molecule RNAs inherent in living organisms and has the function of regulating gene expression at the translational level [163,164,165,166]. miRNA can post-transcriptionally regulate the expression of target genes, thereby playing an essential role in various biological processes, such as proliferation, differentiation, development, and apoptosis. Sun et al. found that miRNA-494 stabilizes HIF-1α expression under normoxic and hypoxic conditions by activating the PI3K/Akt pathway to maintain the normal physiological activity of the hepatocyte lineage in a hypoxic environment, and thus, may be a future therapeutic target for hepatic hypoxia/ischemia injury [167].

### 5.2. Hypoxic Mimetics

#### 5.2.1. Iron Chelator

Over the past two decades, it was recognized that stabilizing HIF-1α to promote the expression of downstream target genes could potentially be applied to the treatment of a range of diseases. However, stabilizing HIF-1α expression by subjecting the entire environment to hypoxia can lead to oxidative stress, which can adversely affect cell metabolism and proliferation; therefore, regulating oxygen content itself to promote HIF-1α expression may not be the ideal form of treatment, as opposed to hypoxic chamber culture [59]. Hypoxia mimetics were created to successfully solve this problem by stabilizing HIF-1α expression under normoxia, which minimizes the potential harm to non-targeted proteins [168]. Hypoxia mimetics can be classified into iron chelators and 2-OG analogs depending on the manner of action. Appropriate doses of Fe^2+^ and 2-OG are required for the induction of HIF hydroxylation by PHD and the hydroxylation of the asparagine residue (N803) in the C-terminal transactivation domain of HIF-1α by FIH. Iron chelators reduce the hydroxylation of proline and asparagine of HIF-1α by strongly binding to Fe^2+^, which reduces the subsequent ubiquitination degradation and dysfunction induced by the inability to bind to CBP/p300 for the formation of the transcriptional complex [169,170]. The most common iron chelators include deferoxamine (DFO) and some specific metal ions, such as cobalt and copper [171,172,173,174,175]. Cobalt ions, especially CoCl_2_, are known to compete with Fe^2+^ in the context of binding to PHD, which stabilizes the expression of HIF-1α to promote angiogenesis and bone regeneration in a normoxic environment. It was shown that the survival of MSCs was not significantly affected when CoCl_2_ was incubated at a concentration of 100 μM for 24–48 h, whereas MSC viability was significantly decreased when CoCl_2_ was incubated at a concentration of 500 μM [176,177]. Copper is an essential trace element in the human body, which can activate the HIF-1α pathway to promote angiogenesis and bone regeneration by inhibiting the hydroxylation of PHD. Zhang et al. prepared graphene oxide–copper nanocomposites (GO-Cu), which were coated on the surface of calcium phosphate cement (CPC) scaffolds to form CPC/GO-Cu scaffolds [178]. GO-Cu was shown to further enhance the expression of VEGF and BMP-2 by activating the HIF-1α pathway and ERK1/2 signaling in rat BMSCs, thereby significantly promoting vascularization and bone regeneration in rat cranial defects. In contrast to metal ions, DFO exhibits localized controlled release, which yields better integration of the drug with bone tissue engineering bio-scaffolds. Zheng et al. prepared a nanoclay-functionalized 3D bioglass scaffold with hypoxia-mimicking properties using DFO, which sustained the release of DFO to induce angiogenesis and bone regeneration by stabilizing the expression of HIF-1α [179]. In addition, Geng et al. fabricated a 3D-printed biodegradable poly(glycerol-co-sebacic acid-co-L-lactic acid-co-polyethylene glycol) (PGSLP)-based scaffold, which was internally filled with gelatin nanofibers, that loaded DFO into the natural scaffold to improve osteogenic function and angiogenesis [180]. In vitro results showed that the scaffold promoted migration and tubular formation of HUVECs by stabilizing the expression of HIF-1α, while in vivo results showed a more complete vascular network and rapid bone repair in a critical-sized rat model containing DFO. Although the available experimental data suggest that DFO does not have significant adverse effects on BMSC activity at concentrations below 120 μM, the short half-life, high cytotoxicity, and off-target effects of DFO compared with the 2-OG analogs limit its further application [181,182].

#### 5.2.2. 2-OG Analogs

The stability and nuclear localization of HIF-1α are controlled by PHD, whereas the binding of HIF-1α to the CBP/p300 cofactor in the nucleus is controlled by FIH [33,183]. This characteristic means that stable expression of HIF-1α could be achieved by regulating PHD and FIH activation. The 2-OG analogs compete with 2-OG to bind to vital binding sites in FIH and PHD, resulting in the inactivation of FIH and PHD to promote the stable expression of HIF-1α. Dimethyloxalylglycine (DMOG) is a common 2-GO analog for which available data show no significant cytotoxicity at concentrations below 5 mm [184]. DMOG, which is less toxic and more biocompatible than DFO, is a cell-permeable small molecule that mimics 2-GO to inhibit both PHD and FIH activity, stabilizing and upregulating HIF-1α levels under normoxic conditions [185]. Although both Fe^2+^ and 2-OG are necessary cofactors for PHD and FIH to exert their functions in inhibiting HIF-1α, PHD and FIH are not equally sensitive to iron chelators and 2-GO analogs in cells [71,186]. While concentrations of iron chelators achieved significant inhibition of prolyl hydroxylation of HIF-1α, they were much less effective in inhibiting asparagine-based hydroxylation of HIF-1α, while metal ions, such as Co(II), were almost entirely ineffective. In contrast, the 2-OG analog DMOG was more effective than iron chelators in inhibiting asparagine hydroxylation of HIF-1α, which may indicate that PHD is more sensitive to the inhibitory effect of iron chelators than FIH, whereas FIH is more sensitive to the inhibitory effect produced by 2-OG analogs. Indeed, high levels of HIF-1α transcription can compensate for the reduced expression of HIF-1α due to PHD2-mediated hydroxylation. Yujie Ha et al. used 3D-printed scaffolds loaded with DMOG to verify the presence of angiogenesis promoted by the activation of HIF-1α during bone repair. In vivo experiments were performed at 6 weeks with immunofluorescence staining for HIF-1α, CD31, and α smooth-muscle actin (α-SMA) expression [83]. The fluorescence signal of angiogenic markers was higher in the DMOG-loaded group compared with the unloaded group, while quantitative analysis showed that the expression of HIF-1α, CD31, and α-SMA was significantly higher in the DMOG-loaded group than in the other groups, indicating that the release of DMOG did promote the stable expression of HIF-1α and effectively promoted angiogenesis. On the other hand, DMOG was shown to promote the survival and homing of MSCs. It was also shown to induce the expression of pro-angiogenic factors in HUVECs that promote angiogenesis. In addition, DMOG, which showed excellent immunomodulatory properties, could induce the polarization of M2 macrophages through different vectors in bone tissue engineering [187,188,189]. However, several studies showed that DMOG reduces RUNX2, ALP, and COL1α1 expression, which decreases the osteogenic potential of BMSCs, as well as osteoblasts [190]. These contradictory findings may be related to impaired cell viability due to the long-term treatment of DMOG. Therefore, we should focus more on controlling the duration of hypoxia simulation in the future to achieve optimal results. Promising specific inhibitors are being investigated to improve the off-target phenomenon of existing hypoxia mimetics, such as the novel 2-GO analogs FG4592, FG2216, and GSK1278863, which have been approved for the treatment of anemia in patients with clinical chronic kidney disease (CKD) and have the advantages of being highly efficient in small amounts and being less toxic [191]. Because they have been approved for clinical use for a relatively short time, only a few studies show their role in the bone formation process. Therefore, we still have a long way to go in further clarifying the effect of these drugs on bone regeneration, but it cannot be denied that hypoxia mimetics provide another new idea to solve the problems in bone regeneration processes.

### 5.3. Others

**Figure 7:**
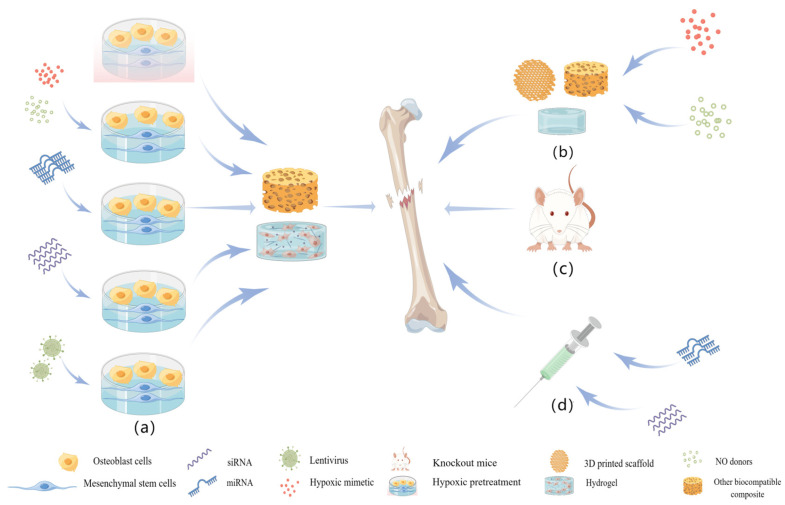
A potential strategy for bone repair with the HIF-1α signaling pathway contains the following: (**a**) loading of cells into hydrogels or other scaffold materials after the stabilization of intracellular HIF-1α via hypoxic preconditioning with 1–5% O_2_ of seeded cells, hypoxic mimetic, gene therapy, and HIF-conducted lentivirus; (**b**) direct loading of a drug with stable HIF-1α into bone tissue engineering scaffolds; (**c**) gene-deficient mice; and (**d**) local delivery of siRNAs or miRNA targeting PHD2 promotes the activation of the HIF-1α signaling pathway.

In addition to the genetic engineering and hypoxia mimetics mentioned above, hypoxic pretreatment and nitric oxide (NO) can also regulate HIF-1α expression, and there are even some purely biomaterial-based approaches that do not rely on any drugs and growth factors (shown in Figure 7) [80,192]. Hypoxic preconditioning of seeded cells by specific media is also a common approach. Luo et al. achieved hypoxic preconditioning by culturing cells in an anaerobic chamber for 48 or 72 h (oxygen concentration at 1%) to promote bone marrow MSC survival by inducing HIF-1α [193]. Zhang et al. performed hypoxic pretreatment of BMSCs by combining 1% O_2_ and 0.5 mM DMOG [194], and this specific hypoxic pretreatment was divided into three steps. First, third-generation BMSCs were exposed to fresh complete medium supplemented with 0.5 mM DMOG at 21% O_2_ for 48 h. Second, third-generation BMSCs were exposed to fresh medium supplemented with 0.5 mM DMOG at 1% O2 for 48 h. Finally, third-generation BMSCs were exposed to a fresh complete medium supplemented with 0.5 mM DMOG at 1% O_2_ for 48 h. The experimental results showed that BMSCs pretreated with hypoxia improved the angiogenic and osteogenic activities and survival of BMSCs under hypoxic conditions by stabilizing the expression of HIF-1α in the cells compared with BMSCs without pretreatment, which provides favorable evidence for further applications of HIF-1α in bone repair.

In addition, NO can stabilize HIF-1α expression by nitrosylating the oxygen-dependent degradation structural domain (ODDD) of HIF-1α, which blocks VHL binding and the subsequent ubiquitination of HIF-1α [195]. In addition, NO donors can play similar roles, such as (±)-S-Nitroso-N-acetylpenicillamine (SNAP) and GSNO. SNAP, which is a nitric oxide donor that prevents the degradation of HIF-1α and promotes its stable expression, can block the interaction between VHL and HIF-1α and inhibit the hydroxylation of HIF asparagine by FIH in the presence of Fe^2+^ [196]. GSNO can further induce HIF-1α expression by inhibiting the activity of PHD and FIH by blocking the binding of Fe^2+^ to the active site of PHD or FIH [197,198].

With the development of material science and biology, some unique purely biomaterial-based approaches can also achieve the effect of simulating hypoxic environments via sequestering iron ions to stabilize HIF-1α expression without the addition of a hypoxic simulant. Sun et al. successfully developed an injectable poly(glycerol sebacate)-co-poly(ethylene glycol)/polyacrylic acid (PEGS/PAA) hydrogel that can simulate hypoxic environments by effectively chelating iron ions through the carboxyl functional group of itself to stabilize HIF-1α expression without the addition of hypoxic mimetics [199].

## 6. Outlook

HIF-1α, which is one of the critical regulators of chondrocyte template formation, is stably expressed in a hypoxic environment, which sustains the survival of cells by altering cellular metabolism. HIF-1α was shown to largely contribute to the construction of vascular networks during bone repair, but the direct relationship between HIF-1α and osteoblast and osteogenic activity remains controversial [114,200]. Prolonged severe hypoxia was demonstrated to have severe adverse effects on bone formation and even a series of brutal outcomes, such as necrosis, which is in line with conventional understandings of cellular activity. Therefore, we put more emphasis on controlled short-term HIF-1α expression. For example, we can achieve a strategy of controlled HIF expression by binding hypoxia mimetics to a biological scaffold by region or gradient for controlled release. The consequential question concerns how to define the “short-term” boundary and control the amount of expression, which is one of the critical questions we will explore in the future. The approach of inducing vascularization by stabilizing HIF-1α expression to initiate ECO in the early stages of bone repair, laying the foundation for future bone hard tissue formation, and then transitioning to IMO at the optimal time for rapid osteogenic activity seems to offer a more promising approach to the treatment of significant bone defects. In addition, we cannot deny some conflicting experimental findings, which may be related to factors such as the degree, frequency, duration of hypoxia, and age of the study subjects. Therefore, a more specific categorization of these influencing factors is needed in the future to make the experimental results more comparable. On the other hand, hypoxia mimetics showed a wide range of applications in bone tissue engineering, but there is a need to focus on the possible side effects of such drugs due to the short period in which they have been introduced into bone tissue engineering. In the future, we should further improve the target specificity and safety of making hypoxia mimetics and explore the exact mechanism by which they promote bone regeneration. In addition, the hypoxic environment, which is one of the main features of tumors, maintains the over-supported vascular system required for tumor growth. HIF-1 and HIF-2 are involved in tumor angiogenesis, which is closely associated with the rapid proliferation and metastasis of tumor cells, especially HIF-2, which plays an important role in the rapid deterioration of tumors. Therefore, more relevant studies are needed in the future to further identify the complex biological effects of HIFs in bone repair and tumors under hypoxic conditions, as well as their molecular mechanisms. How to make optimal use of HIFs to guide bone regeneration while avoiding tumor recurrence and growth is vital for the application of HIFs in clinical treatment in the future. Nevertheless, the role of the stable expression of HIF-1α in the bone regeneration process remains a research direction with great potential.

## Data Availability

Not applicable.
